# Lithium-Induced Hyperparathyroidism in a Patient With Bipolar Disorder: The Importance of Monitoring Complications in Patients Undergoing Chronic Lithium Therapy

**DOI:** 10.7759/cureus.80030

**Published:** 2025-03-04

**Authors:** Edgar Dehesa-Lopez, Mónica Fernanda Curiel-González, Estefanía Araujo-Rocha

**Affiliations:** 1 Nephrology, Universidad Autonoma de Sinaloa, Culiacán, MEX; 2 Nephrology, Hospital Civil de Culiacán, Culiacán, MEX; 3 Nephrology, Centro de Investigación y Docencia en Ciencias de la Salud, Culiacán, MEX; 4 Nephrology, Universidad Autónoma de Sinaloa, Culiacán, MEX

**Keywords:** lithium-associated complications, lithium-induced hypercalcemia, lithium-induced hyperparathyroidism, lithium-induced nephrotoxicity, lithium-related endocrine problems

## Abstract

Lithium remains a cornerstone in the pharmacological management of bipolar disorder due to its well-established mood-stabilizing properties. However, chronic lithium therapy is associated with a spectrum of potential complications, including hyperparathyroidism and progression to chronic kidney disease (CKD). We present the case of a patient with bipolar disorder who developed lithium-induced hyperparathyroidism and CKD following long-term lithium therapy. During a routine follow-up, the patient was found to have hypercalcemia, elevated parathyroid hormone levels, and increased serum creatinine. A comprehensive diagnostic workup confirmed the diagnosis of lithium-induced hyperparathyroidism and CKD. Notably, the patient's condition improved following the discontinuation of lithium therapy. This report details the patient's clinical course, diagnostic evaluation, and management strategies, underscoring the importance of routine monitoring of parathyroid function (serum calcium, PTH levels) and renal function (serum creatinine, electrolytes, and urinalysis) in patients undergoing long-term lithium treatment. Early detection and intervention are critical to mitigating the risk of these complications and optimizing patient outcomes.

## Introduction

Bipolar disorder is a chronic mental illness that encompasses various disorders of emotion, energy, and thought, characterized by the presence of biphasic mood episodes: depression and either mania (bipolar I) or hypomania (bipolar II) (1)[1]. It is estimated that bipolar disorder affects more than 1% of the global population and has been linked to an increased risk of suicide, with an annual rate ranging from 0.4% to 1.4% [1,2]. Lithium has been regarded as the cornerstone of treatment for bipolar disorders for decades due to its effectiveness in reducing the risk of suicide [3]. However, it has been documented that patients who use lithium chronically may experience several side effects, such as hyperparathyroidism, hypothyroidism, weight gain, nephrogenic diabetes insipidus, and chronic kidney disease (CKD) [4].

The prevalence of lithium-induced hyperparathyroidism (Li-HPP) has not been well established due to its asymptomatic nature in most cases and the lack of routine measurement of PTH and calcium levels in these patients. A prevalence rate of 2.7% (7.5% higher than that of the general population) for Li-HPP and 3.6% for persistent hypercalcemia has been reported in patients treated with lithium for 15 or more years [5]. On the other hand, it has been documented that these patients exhibit serum PTH and calcium levels up to 10% higher than control patients [6].

The clinical consequences of Li-HPP are numerous and include renal stones, nephrocalcinosis, osteoporosis, osteopenia, worsening psychiatric conditions, dyspepsia, dysrhythmias, hypertension, dehydration, and renal impairment [7]. Therefore, monitoring these complications is crucial in the management of these patients.

This case highlights the importance of early detection and intervention for lithium-induced hyperparathyroidism to prevent irreversible complications in patients with bipolar disorders undergoing chronic lithium treatment.

## Case presentation

This report describes a 63-year-old female with a history of hypertension diagnosed one year ago, managed with losartan 50 mg daily. She also has dyslipidemia, treated with atorvastatin 20 mg and bezafibrate 200 mg daily, and urinary incontinence, treated with oxybutynin 10 mg daily. She was diagnosed with bipolar disorder 30 years ago and has been on long-term lithium therapy (300 mg twice daily) and olanzapine (10 mg daily) with a sustained clinical response and currently without symptoms.

The patient was referred to the nephrology clinic for further evaluation of chronic kidney disease, which was recently diagnosed during an internal medicine consultation. During her initial visit, she reported being asymptomatic and denied the use of nephrotoxic medications. She did not exhibit symptoms of polyuria or polydipsia and indicated that her blood pressure was well-controlled at home. The patient reported having irregular monitoring of serum lithium levels during her treatment, with recent serum lithium levels at 0.4 mmol/L (recommended therapeutic range 0.5-0.8 mmol/L). Laboratory tests revealed elevated serum creatinine level, decreased estimated glomerular filtration rate (eGFR), hypercalcemia, hypermagnesemia, normal phosphorus levels, and a urinalysis without proteinuria (Table 1).

**Table 1:** Initial laboratory of the patient. eGFR = estimated glomerular filtration rate

Parameters	Values	Reference values
Parathyroid hormone (pg/mL)	112.9	10-65
Creatinine (mg/dL)	1.6	0.7-1.1
Urea (mg/dL)	56	10-50
eGFR (ml/min/m2SC)	36	80-120
Hemoglobin (g/dL)	13.6	11-15
Leucocytes (103/ µL)	10.3	4.5-10
Platelets (103/ µL)	255	150-300
Na (mEq/L)	138	135-145
K (mEq/L)	4	3.5-5.0
Cl (mEq/L)	108	98-106
Ca (mg/dL)	10.7	8.5-10.2
P (mg/dL)	3	2.5-4.5
Mg (mg/dL)	2.4	1.7-2.2

**Figure 1:**
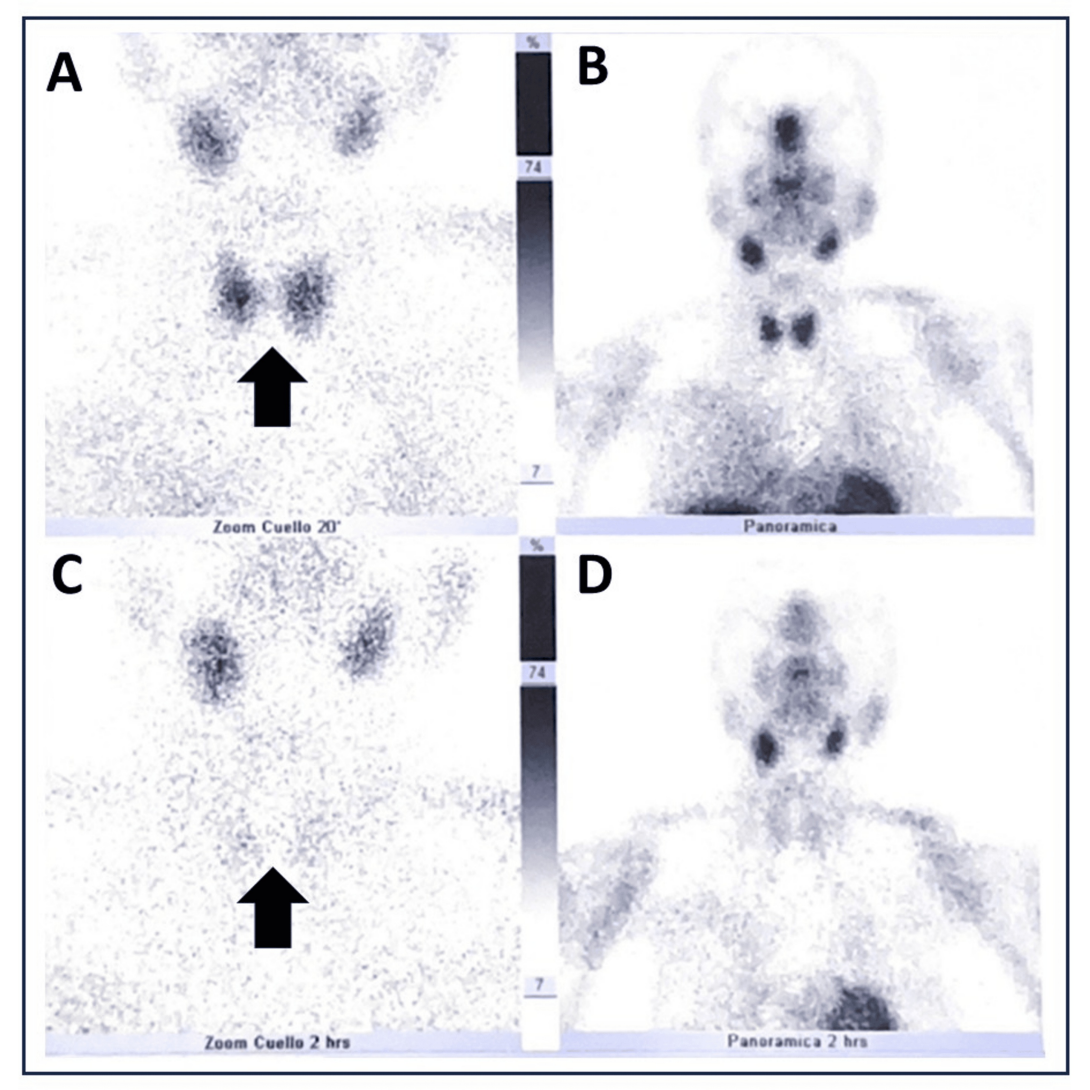
Parathyroid scintigraphy A dose of 20 mCi of 99mTc-MIBI is administered intravenously, and static images are acquired at 20 and 120 minutes in the neck region. Early static image at 20 minutes (panels A and B): The thyroid gland shows homogeneous distribution, with no abnormal focal uptake (arrow in panel A) . Early static image at 120 minutes (panels B and C): No abnormal focal uptake is observed, in addition to the typical biodistribution of the radiopharmaceutical (arrow in panel C).

A diagnosis of CKD was established, classified as KDIGO G3bA1, likely associated with long-term lithium use. Due to persistent hypercalcemia observed in previous laboratory tests, parathyroid hormone (PTH) levels were measured. On follow-up, persistent hypercalcemia was documented, with elevated serum PTH levels. In this clinical context, hyperparathyroidism associated with chronic lithium use was suspected. A parathyroid scintigraphy was performed to rule out primary hyperparathyroidism, which was negative for parathyroid adenomas (Figure 1).

**Figure 2:**
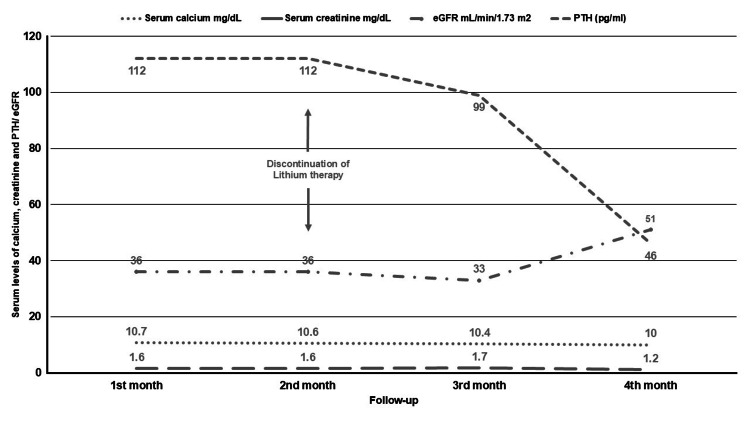
Changes in serum calcium, PTH, serum creatinine, and eGFR following lithium discontinuation. PTH = parathyroid hormone; eGFR = estimated glomerular filtration rate

Furthermore, a 24-hour urinary calcium excretion test decreased with 54 mg (normal range 100-250 mg/day), and albuminuria was recorded at 5 mg/day. Bone densitometry showed a lumbar T-score of -2.6 SD and a total hip T-score of -2.3 SD, indicating low fracture risk as assessed by the FRAX score, which was calculated to be 5.4%. Consequently, a diagnosis of Li-HPP was confirmed. The patient's pharmacological treatment was adjusted, with the gradual withdrawal of lithium over a two-week period and the initiation of valproic acid (200 mg twice daily) under psychiatric supervision. Figure 2 illustrates the evolution of serum calcium and PTH levels following lithium discontinuation. A gradual decline and subsequent normalization of both parameters were observed four months after discontinuing lithium therapy. Regarding her bipolar disorder, the patient remained asymptomatic since the cessation of lithium, with treatment consisting of valproic acid (200 mg twice daily) and olanzapine (10 mg daily).

In relation to renal function, a significant improvement in the eGFR from 36 ml/min to 51 ml/min was observed following the discontinuation of lithium therapy and normalization of serum calcium levels (Figure 2).

## Discussion

Lithium remains the most efficacious therapy for a significant proportion of patients with type 1 bipolar disorder, protecting against both depression and mania, as well as being the only therapy known to reduce the risk of suicide in this patient population [3,8]. However, despite the demonstrated therapeutic benefits of its use, chronic lithium therapy has been associated with severe adverse effects on the gastrointestinal, endocrine, and renal systems [6,7,9].

Patients treated with lithium exhibit a 10% increase in calcium and PTH levels compared to control subjects, along with an elevated risk of developing hypercalcemia [6,10]. In addition, lithium-treated patients display a high proportion of elevated PTH (8.6%) and calcium levels (24.1%), as well as a higher prevalence of hyperparathyroidism (18%) in comparison with subjects who had never been exposed to lithium [10,11].

The true incidence of lithium-induced hyperparathyroidism remains unclear, as PTH levels are not routinely assessed in these patients, and many cases are asymptomatic. Bann et al. reported that only 1.9% of 4,917 patients on chronic lithium therapy had a PTH determination during follow-up, and even among those with documented hypercalcemia, only 17.2% had a PTH measurement [12].

**Figure 3:**
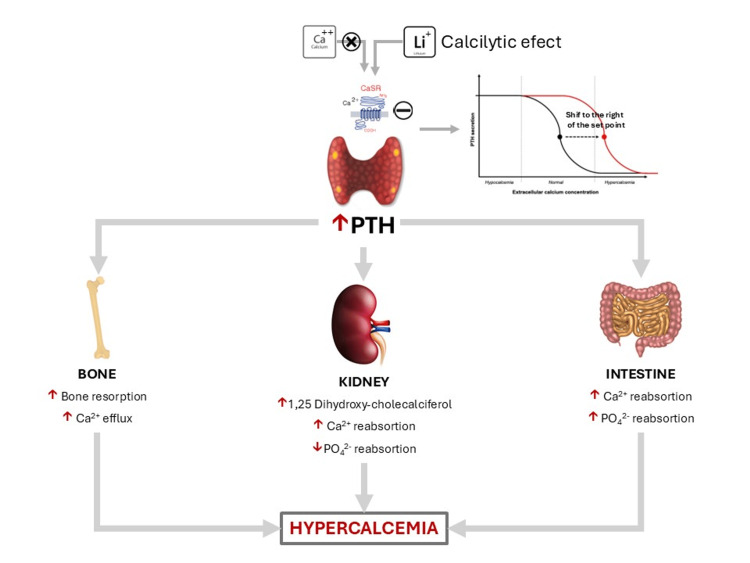
Pathophysiology of lithium-induced hyperparathyroidism PTH = parathyroid hormone, CaSR = calcium sensing receptor

The exact mechanism underlying this endocrine complication has not been fully established. The most accepted hypothesis is that lithium increases the secretion of PTH, and this increase in secretion is associated with hyperplasia of the four parathyroid glands. This occurs because ionized lithium (Li+) resembles ionized calcium (Ca++), acting as a calcilytic by antagonizing the calcium-sensing receptor (CaSR) [7,10]. As a result, the receptor perceives plasma calcium levels as potentially low, leading to increased secretion of PTH, which subsequently drives calcium release from the bones and increases calcium reabsorption in the kidneys, as well as calcium absorption in the intestine (Figure 3). When these PTH levels remain elevated in response to chronic lithium use, the four parathyroid glands generally develop hyperplasia over the years. This increase in size and function can lead to autonomous secretion of PTH, as the glands no longer respond to changes in plasma calcium levels [10].

The clinical presentation of lithium-induced hyperparathyroidism includes multiple symptoms and clinical manifestations, which encompass bone pain, fractures, kidney stones, and gastrointestinal disturbances [7,13]. It is noteworthy that symptoms can vary by patient age, with patients over 65 years more likely to be asymptomatic or to present with osteoporosis, while younger patients predominantly exhibit nephrolithiasis and fibrous osteitis [14]. In cases where serum calcium levels exceed 12 mg/dL, patients are more likely to experience symptoms associated with hypercalcemia, which include anorexia, nausea, vomiting, constipation, altered mental states, dehydration, polyuria, and polydipsia [15]. In addition, persistent hyperparathyroidism, along with the consequent hypercalcemia, can have detrimental effects on bone mineral metabolism, urinary excretion, and blood vessels, as well as exacerbating psychiatric manifestations, including memory loss, disturbances of consciousness, sleep alterations, fatigue, and depression, as well as manic, psychotic, and obsessive-compulsive symptoms [14].

The diagnosis of Li-HPP is confirmed by the presence of elevated levels of PTH, serum calcium, and magnesium [12]. This condition often exhibits characteristics that are distinct from primary hyperparathyroidism. These include serum calcium levels that range from slightly to significantly elevated, PTH levels that are high-normal to elevated, normal phosphate levels, hypermagnesemia, and hypocalciuria. By contrast, primary hyperparathyroidism typically presents with more pronounced elevations in calcium and PTH levels, low serum phosphate levels, normal magnesium levels, and hypercalciuria. On the other hand, hypercalcemia and accompanying symptoms in Li-HPP typically resolve upon discontinuation of therapy. However, this outcome is not universally observed, as some authors have reported persistent symptoms even after stopping treatment. Notably, hypercalcemia may not improve within eight weeks of discontinuing lithium therapy [5]. Another key difference is observed in bone densitometry, where mild osteopenia or normal bone density is seen in cases of Li-HPP. By contrast, primary hyperparathyroidism is typically an indolent disease. Nevertheless, certain individuals may present with osteoporosis or, in rare instances, fibrous osteitis [16].

The cornerstone of management for lithium-induced hyperparathyroidism consists of discontinuing lithium therapy and transitioning to an alternative treatment, if available, along with monitoring serum calcium and PTH levels [9]. However, in situations where hyperparathyroidism persists or complications such as kidney stones or osteoporosis arise, surgery may be required [13].

In our patient, the diagnosis of CKD KDIGO G3bA1, likely secondary to lithium nephropathy, was also established. The risk of developing CKD in patients on long-term lithium treatment has now been well established by several clinical, histopathological, and epidemiological studies [9]. A recent retrospective study in France by Boivin et al. examined 248 patients treated with long-term lithium therapy and reported a decrease in renal function under lithium therapy estimated at -2.9 mL/min/year. The deterioration of renal function was consequently two to three times more rapid compared to the general population, where the average annual decline in GFR is estimated to be approximately 1 mL/min/1.73 m² [17]. The progression of lithium-induced CKD is slow, requiring a period of 10 to 20 years to progress to end-stage renal failure [9]. Various studies estimate the prevalence of CKD among patients undergoing lithium treatment to be between 10% and 35% [18]. The incidence of end-stage renal disease (ESRD) attributed to lithium is very low, estimated to be between 0.2% and 0.7%; however, this still represents an almost eightfold increased risk of ESKD compared to the general population [9]. Conversely, there is a higher incidence of CKD stage 3 in patients on long-term lithium therapy compared to the general population, with 21-55% of long-term lithium users falling into this category [9].

The risk of developing lithium nephropathy is multifactorial, involving genetic predisposition, lithium-specific factors (duration of lithium therapy, dosage and type of drug used, serum levels, and episodes of acute lithium intoxication), female gender, age, concurrent use of nephrotoxic medications, hypertension, diabetes, nephrogenic diabetes insipidus, and lower initial glomerular filtration rate [9,19]. In addition, the degree of interstitial fibrosis observed in renal biopsy and the presence of heavy proteinuria have been associated with a poor prognosis in the progression of lithium nephropathy [19].

The most critical therapeutic decision in a patient with lithium nephropathy is determining the optimal time to discontinue lithium treatment. The risk of relapse in a clinically stable patient, along with the potentially increased risk of suicide with alternative treatments, must be carefully balanced against the risk of progressing to ESRD. Discontinuation at an earlier stage of CKD may be appropriate if the mood disorder has been stable, an alternative effective therapeutic option is available for the patient, and there is clear evidence of a persistent decline in renal function over time with no other identifiable cause [9]. Various studies have reported that renal function either remains stable or improves after discontinuing lithium. However, other studies have shown that a subset of patients with an eGFR < 40 ml/min continue to experience renal function deterioration despite the discontinuation of lithium; therefore, the decision to suspend lithium therapy should be carefully evaluated by both the psychiatrist and nephrologist [9,20].

Clinical practice guidelines recommend that all patients on chronic lithium therapy undergo baseline and subsequent serum calcium measurements every three to six months, as well as serum lithium level determinations every three to six months, with a recommended therapeutic range of 0.5-0.8 mmol/L [4]. If persistent hypercalcemia is diagnosed during follow-up, further diagnostic evaluation is warranted, including PTH measurement to rule out lithium-induced hyperparathyroidism [10].

For renal function monitoring, baseline and subsequent measurements of serum creatinine and eGFR every three to six months are recommended. Referral to a nephrologist is advised for patients with progressive renal function decline, particularly if the decline exceeds 4 ml/min/year, for patients with an eGFR of less than 30 ml/min, for those with suspected alternative etiologies of renal damage (such as hematuria or proteinuria), or for patients already presenting with complications associated with CKD [4,9]. 

## Conclusions

Li-HPP is a potential complication in patients with bipolar disorder undergoing long-term lithium therapy. This condition is associated with significant clinical consequences, including hypercalcemia, osteoporosis, renal stones, and impaired renal function. Active screening, involving the evaluation of endocrine function (PTH, thyroid-stimulating hormone, and serum calcium levels), renal function (serum creatinine, electrolytes, and urinalysis), and serum lithium levels during treatment, constitutes a critical tool for the prevention, early diagnosis, and timely management of this complication. In most cases, Li-HPP resolves following the discontinuation of lithium. However, discontinuation should be carried out under the supervision of a multidisciplinary team, with careful evaluation of the balance between the patient's psychiatric stability and the risk of complications.
